# Cannabis-assisted psychotherapy for complex dissociative posttraumatic stress disorder: A case report

**DOI:** 10.3389/fpsyt.2023.1051542

**Published:** 2023-02-09

**Authors:** Anya Ragnhildstveit, Miriam Kaiyo, Matthew Brian Snyder, Laura Kate Jackson, Alex Lopez, Chasity Mayo, Alyssa Claire Miranda, River Jude August, Paul Seli, Reid Robison, Lynnette Astrid Averill

**Affiliations:** ^1^Integrated Research Literacy Group, Draper, UT, United States; ^2^Department of Psychiatry, University of Cambridge, Cambridge, United Kingdom; ^3^Department of Psychology and Neuroscience, Duke University, Durham, NC, United States; ^4^Department of Family and Consumer Studies, University of Utah, Salt Lake City, UT, United States; ^5^Consciousness and Transformative Studies, National University, San Diego, CA, United States; ^6^Numinus Wellness, Draper, UT, United States; ^7^Department of Psychiatry, University of Utah School of Medicine, Salt Lake City, UT, United States; ^8^Menninger Department of Psychiatry and Behavioral Sciences, Baylor College of Medicine, Houston, TX, United States; ^9^Michael E. DeBakey Veterans Affairs Medical Center, Houston, TX, United States; ^10^Department of Psychiatry, Yale School of Medicine, New Haven, CT, United States; ^11^Department of Veterans Affairs, Clinical Neuroscience Division, National Center for PTSD, West Haven, CT, United States

**Keywords:** cannabis, medicinal cannabis, cannabis-assisted psychotherapy, dissociation, posttraumatic stress disorder, trauma, treatment, case report

## Abstract

**Background:**

A dissociative subtype of posttraumatic stress disorder, known as “D-PTSD”, has been included in the Diagnostic and Statistical Manual of Mental Disorders, Fifth Edition. In addition to meeting criteria for PTSD, patients endorse prominent dissociative symptoms, namely depersonalization and derealization, or detachment from one's self and surroundings. At present, this population is supported by a highly heterogeneous and undeveloped literature. Targeted interventions are therefore lacking, and those indicated for PTSD are limited by poor efficacy, delayed onset of action, and low patient engagement. Here, we introduce cannabis-assisted psychotherapy (CAP) as a novel treatment for D-PTSD, drawing parallels to psychedelic therapy.

**Case presentation:**

A 28-year-old female presented with complex D-PTSD. In a naturalistic setting, she underwent 10 sessions of CAP, scheduled twice monthly over 5 months, coupled with integrative cognitive behavioral therapy. An autonomic and relational approach to CAP was leveraged, specifically psychedelic somatic interactional psychotherapy. Acute effects included oceanic boundlessness, ego dissolution, and emotional breakthrough. From baseline to post-treatment, the patient showed a 98.5% reduction in pathological dissociation, as measured by the Multidimensional Inventory of Dissociation, no longer meeting criteria for D-PTSD. This was accompanied by decreased cognitive distractibility and emotional suffering, as well as increased psychosocial functioning. Anecdotally, the patient has sustained improvements for over 2 years to date.

**Conclusions:**

There is urgency to identify treatments for D-PTSD. The present case, while inherently limited, underscores the potential of CAP as a therapeutic option, leading to robust and sustained improvement. Subjective effects were comparable to those produced by classic and non-classic psychedelics, such as psilocybin and ketamine. Further research is warranted to explore, establish, and optimize CAP in D-PTSD, and to characterize its role in the pharmacological landscape.

## Introduction

Posttraumatic stress disorder (PTSD) is a chronic and disabling psychiatric condition. It has an estimated lifetime prevalence of 7.7% in the United States (1, 2), with a 12-month prevalence rate of 4.1% (1, 3). While presentations vary, PTSD is characterized by thought intrusion, persistent avoidance, negative mood and cognition, and alterations in arousal and reactivity (4). These symptoms are associated with trauma exposure, including sexual, interpersonal, and organized violence, that may accumulate with repeat events over time (5). This leads to considerable psychosocial and occupational disability, with negative downstream effects on quality of life.

Most recently, a dissociative subtype of PTSD, known as “D-PTSD”, has been included in the Diagnostic and Statistical Manual of Mental Disorders, Fifth Edition [DSM-5; (4)]. Apart from meeting criteria for PTSD, these patients endorse prominent dissociative symptoms, namely depersonalization and derealization, or detachment from one's self and surroundings, as well as emotional disengagement (4, 6). Other complaints include memory disturbance, gaps in awareness, and sensory illusions (7, 8). A recent meta-analysis estimated the prevalence of D-PTSD as 38.1% in patients with PTSD (9). The phenotype is further linked to increased role impairment, psychiatric comorbidity, and suicide risk compared to PTSD alone (10, 11).

To date, only two medications are approved by the Food and Drug Administration for PTSD, sertraline and paroxetine, both of which are selective serotonin reuptake inhibitors (SSRIs). Even when optimally delivered, up to 60% of patients do not respond to SSRIs and <30% achieve remission (12–14). This often results in early medication withdrawal. Moreover, the latency period of these slow-acting antidepressants significantly elevates the risk of suicide and self-injurious behavior (15, 16). As such, trauma-focused psychotherapies are designated as first-line treatments for PTSD, including prolonged exposure (PE) and cognitive processing therapy (CPT) (17). However, these interventions are limited by high attrition rates (>45%) and low patient engagement (18). Novel strategies are therefore urgently needed, especially those targeting dissociative symptoms.

Cannabis, colloquially referred to as “marijuana,” is derived from the *Sativa* and *Indica* species of the Cannabis plants (19). It contains cannabinoids and several other chemicals acting on cannabinoid type-1 (CB_1_) and type-2 (CB_2_) receptors in neurons and immune cells (20). This includes Δ^9^-tetrahydrocannabinol (THC), the main psychoactive ingredient of cannabis known to produce alterations in perception, awareness, and insight; or the “high” commonly reported by users. THC also has analgesic, antiinflammatory, and antioxidant properties (21). In contrast, cannabidiol (CBD) is a non-psychoactive constituent, with anxiolytic, antipsychotic, and anticonvulsive effects (21). Evidence on the clinical benefits of cannabis has thus been growing for a host of indications, ranging from pain to neurologic to sleep disorders (22, 23). There is a particular interest in its application to psychiatric conditions, including PTSD (24). While studies have yet to show robust improvements, their results are confounded by various factors, such as underpowered sample sizes, heterogeneous populations, variant dosing regimens, and anecdotal reporting (25–27). Here, we present the first case, to the best of our knowledge, of cannabis-assisted psychotherapy (CAP) as a treatment for complex D-PTSD, in accordance with CARE (CAse REport) guidelines (28).

## Case presentation

A 28-year-old female presented with medical, physical, and sexual polytrauma. At 6 months of age, she was diagnosed with hip dysplasia, resulting in 14 corrective surgeries by age 7. This was compounded by scoliosis and associated chronic pain. The patient was maternally neglected, frequently left without care, adequate food, and supervision. At age 9, she was sexually abused, on multiple occasions, by her mother's boyfriend. This intensified through repeat sexual abuse by a high school partner, persisting until 15 years of age. Overtime, the patient developed a constellation of symptoms, including excessive fear, debilitating anxiety, and negative affect.

According to psychiatric records, she was diagnosed with PTSD plus comorbid anxiety and major depression. The patient first partook in cognitive behavioral therapy (CBT), followed by internal family systems (IFS) therapy and eye movement desensitization and reprocessing (EMDR). These interventions targeted relational trauma, distressing internal experiences, and attachment deficits, respectfully; however, each resulted in poor symptom relief. She was then prescribed various antidepressants, namely sertraline (Zoloft^®^), escitalopram (Lexapro^®^), and bupropion (Wellbutrin^®^). Yet, the patient did not respond to multiple trials of adequate dose and duration. Six years later, she obtained a medical cannabis card to self-manage her chronic pain and PTSD symptoms. This was issued by the Utah Department of Health under the Utah Medical Cannabis Act (House Bill 3001). Despite initial improvement, the patient discontinued use following increased fear and paranoia. Her disease state consequently worsened, leading to agoraphobia and functional disability: “I couldn't leave my house. I couldn't think or clean or make food. I couldn't take care of myself. Everything was too much. Too hard. I was scared all the time, even to use the bathroom at night.” She further reported death anxiety with acute panic attacks: “The thought of dying was overwhelming. It constantly interfered with my daily life.” This decline in mobility and cognition motivated her to re-consider cannabis use, this time with therapy. The patient's prior experience with cannabis, showing signals of improvement in chronic pain and trauma-related symptoms; the market availability and legality of cannabis in the state of Utah; and the accessibility of specialized, clinician-guided services utilizing cannabis, also influenced her decision to seek CAP.

## Diagnostic assessment

Upon intake, the patient underwent the Structured Clinical Interview for DSM-5 (SCID-5) (29), confirming complex PTSD. The Multidimensional Inventory of Dissociation (MID) (30), a 218-item multiscale instrument of dissociative phenomena, was also administered. Diagnostic impressions revealed a dissociative subtype, with clinically significant symptoms (cut-off score > 100) present in all three criteria: A (general PTSD dissociative symptoms), B (partially dissociated intrusions), and C (fully dissociated actions). Other complaints included absent-mindedness, inattention, and emotional distress. As such, her clinician recommended repeat CAP, leveraging psychedelic somatic interactional psychotherapy (PSIP) (31). This approach involves autonomic and relational processing, activated by legally prescribed cannabis, to target index trauma. In the patient's case, using PSIP to target core dissociation and interpersonal trauma, stemming from early abandonment, abuse, and enmeshment. Her resistance to first-line treatment for PTSD, including CBT and EMDR, further justified PSIP as an alternative to established, evidence-based psychotherapeutic techniques.

## Treatment approach

**Figure 1:**
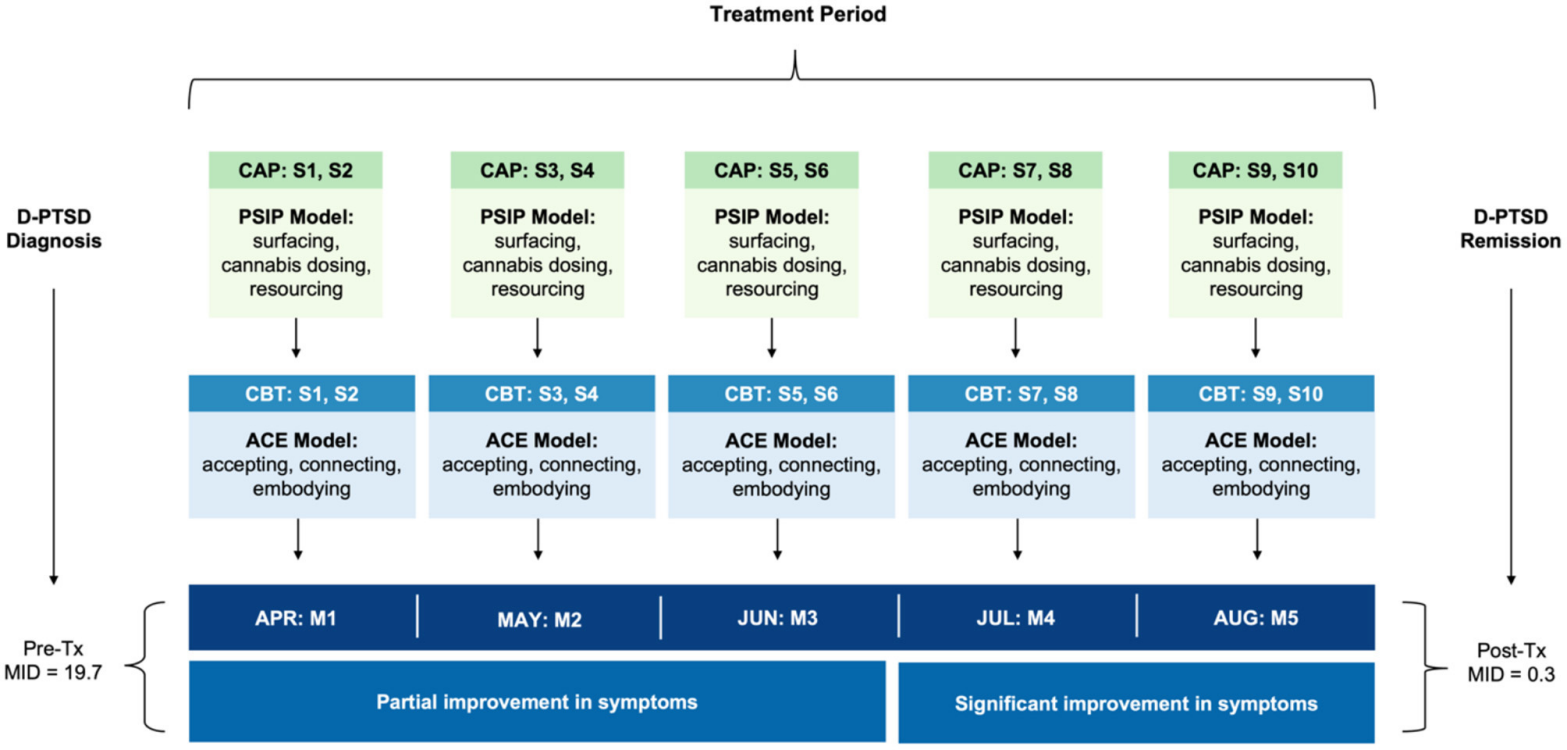
Timeline of clinical events. The patient received 10 CAP sessions, scheduled twice monthly, two-to-three weeks apart, as a treatment for complex D-PTSD. An autonomic and relational approach to psychotherapy was leveraged, namely PSIP. Each CAP session was accompanied by CBT, using the ACE model for integration, to decode experiential phenomena. CBT sessions occurred within 1 week of CAP. ACE, accept, connect, and embody (psychotherapy); CAP, cannabis-assisted psychotherapy; CBT, cognitive behavioral therapy; D-PTSD, dissociative posttraumatic stress disorder; M, month; MID, multidimensional inventory of dissociation; PSIP, psychedelic somatic interactional psychotherapy; S, session; Tx, treatment.

Akin to psychedelic-assisted therapy [reviewed in (32–34)], CAP included preparation, dosing, and integration. The patient received 10 CAP sessions, scheduled twice monthly over 5 months. This frequency was based on treatment response and tolerability (Figure 1). Sessions were followed by integrative CBT, within 1 week of CAP, aimed at decoding experiential phenomena. The “accept, connect, and embody” (ACE) (35) model, predicated on psychological flexibility, was used to facilitate internal and behavioral change. ACE encourages patients to accept challenging experiences, connect to positive material, and deeply attend somatic cues. See Table 1 for details regarding both CAP and CBT regimens. The setting included a private office with warm lighting, mural tapestries, and live plants. All sessions were video recorded for ethical, safety, and integration purposes.

**Table 1:** Psychotherapeutic regimens.

**Tx**	**Freq**	**Min**	**Schedule**	**Approach**	**Emphasis**	**Components**
CAP	10	120	Twice monthly, two-to-three weeks apart, over 5 months	Directive, interactional; PSIP model	Core index trauma	Psychological processing, mind-body grounding, cannabis dosing with therapy
CBT	10	60	Twice monthly, 1 week following CAP, over 5 months	Structured, goal-oriented; ACE model	Treatment integration	Experiential decoding, mindful awareness, meaning-making, insight formation, goal setting

ACE, accept, connect, and embody (psychotherapy); CAP, cannabis-assisted psychotherapy; CBT, cognitive behavioral therapy; Freq, frequency; Min, minute; PSIP, psychedelic somatic interactional psychotherapy; Tx, treatment.

## Cannabis-assisted psychotherapy

**Figure 2:**
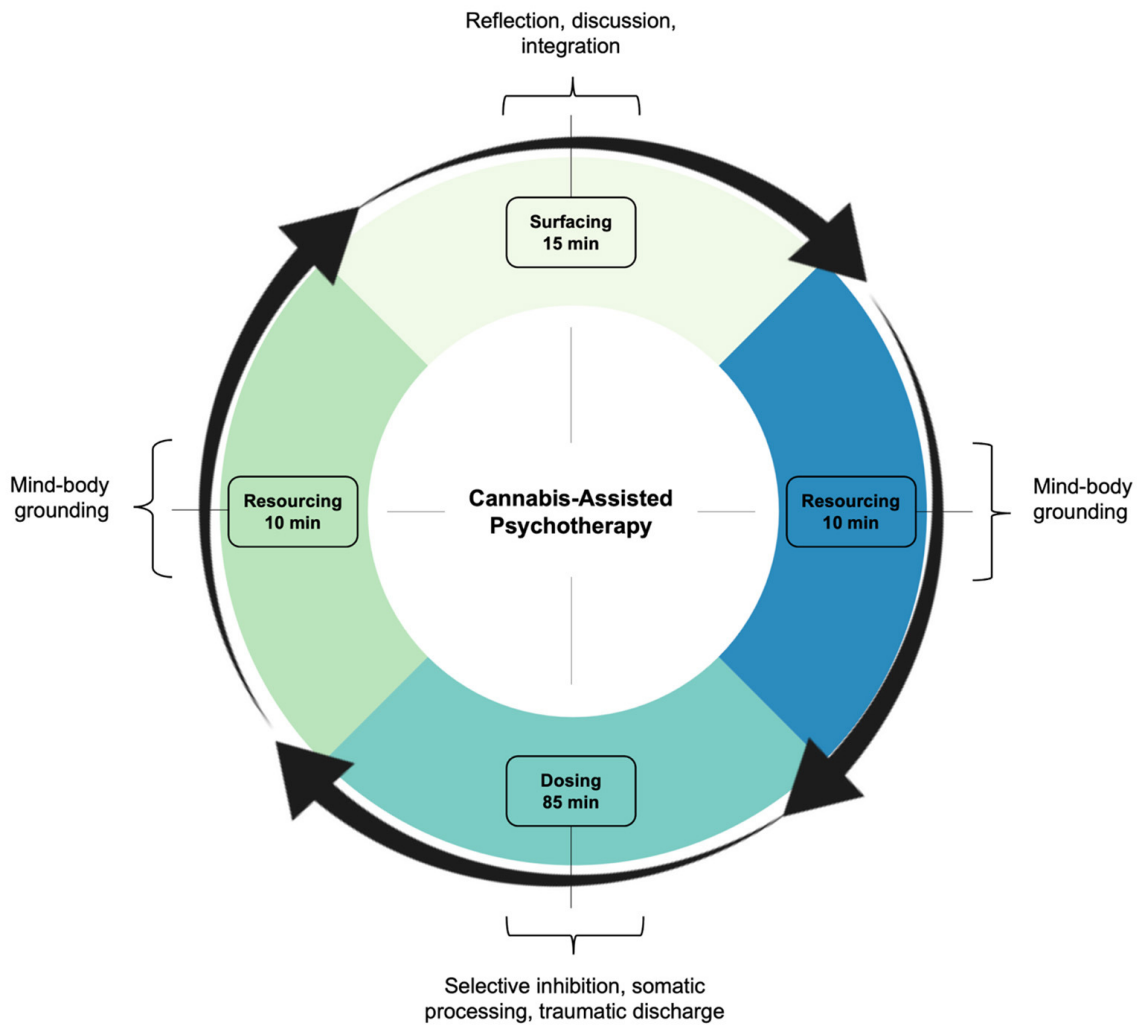
Treatment framework. In line with the PSIP model, CAP involved three criteria: surfacing, resourcing, and dosing. Surfacing readied the patient for treatment, integrating experiences from prior sessions, excluding the first one. Resourcing grounded the patient before and after treatment, comprising mindfulness and nervous system regulation. Dosing included cannabis and psychotherapy, as an interactive treatment, targeting dissociative symptoms and interpersonal trauma. CAP, cannabis-assisted psychotherapy; PSIP, psychedelic somatic interactional psychotherapy.

Sessions were primed with psychoeducation and intention setting. The clinician first discussed D-PTSD and its pathogenesis, explained the course of treatment and possible risks, and taught various grounding and self-regulation techniques. Thereafter, the patient developed a clear and positive motive for CAP, designed to help navigate potentially difficult content. Once primed, sessions began with 10–15 min of “surfacing.” Here, thoughts, emotions, and insights from prior CAP sessions were discussed, excluding the first one. This was followed by 10–15 min of “resourcing,” aimed at achieving a present state of calm. This involved clinician-guided exercises, such as deep breathing, positive memory recall, and imaginative thinking. Dosing subsequently occurred. Using a battery-operated vape pen, the patient inhaled 6–10 mg of cannabis chemotype II, a mixed ratio of THC and CBD. Subjective effects included oceanic boundlessness and ego dissolution (Table 2). The clinician then initiated PSIP, targeting dissociative symptoms. Through selective inhibition, the patient suppressed voluntary movement and coping strategies, while fully acknowledging and experiencing urges. This induced hypo- and hyper-arousal, presenting as depersonalization and psychomotor agitation, respectively. Muscle contractions, increased body temperature, and physical discomfort followed. To promote somatic processing, the patient endured the state until sensations abated. This resulted in “traumatic discharge,” breaking emotional and memory blocks, often terminating in psychocatharsis. Sessions closed with 10–15 min of resourcing, with a return to the present moment (Figure 2). The clinician ended by completing a risk assessment to ensure the patient's safety. Acute psychophysiological changes, albeit their intensity, were generally well tolerated and resolved completely. No adverse events were clinically observed nor self-reported.

**Table 2:** Subjective effects.

**Subjective effects**	**Definitions**	**Patient perspective**
Oceanic boundlessness	Oneness with the universe accompanied by a sense of awe	“I had these moments of insight that were very profound. Everything would come together all at once: fear, beauty, wonder. Sometimes they would ebb and flow during a session too. It was spiritual in a way. I felt enlightened.”
Ego dissolution	Complete loss of subjective self-identity	“Once I started dissociating, I detached from who I was and everything around me. I just dissolved into nothingness. It was dark and very uncomfortable at times.”
Emotional breakthrough	Categorical leap in affective experience	“I had my biggest breakthrough after session six. I was able to connect with my younger self and tell her that this [trauma] wasn't her fault. That she did the best she could. It was an emotional purging. After that, I started seeing everything in a new light, and started forgiving and accepting myself and past experiences. That's when my healing truly began.”

In response to treatment, the patient reported changes in three facets of mysticism: Oceanic boundlessness, ego dissolution, and emotional breakthrough.

**Figure 3:**
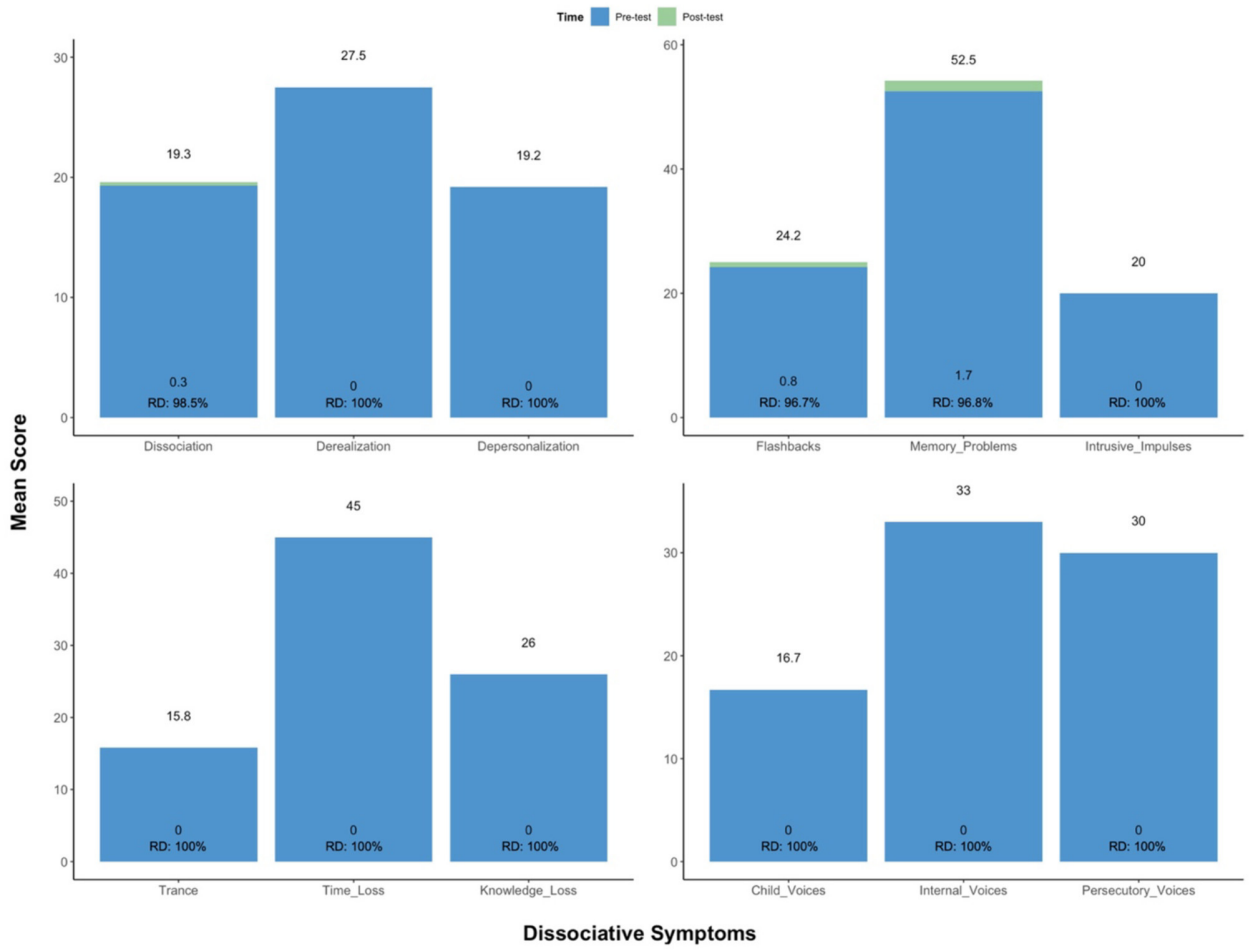
Change in scoring on the MID. From baseline to post-treatment, following all CAP and CBT sessions, the patient showed a robust improvement in clinically significant dissociative symptoms, no longer meeting criteria for D-PTSD. CAP, cannabis-assisted psychotherapy; D-PTSD, dissociative posttraumatic stress disorder; MID, multidimensional inventory of dissociation; RD, reduction.

Follow-ups occurred 1 day post-CAP *via* telephone. Between sessions, the patient watched the previous recording, journaled insights revealed by the experience, and engaged in CBT. This was considered integration work. Her symptoms partially remitted over the first six sessions, and significantly remitted thereon out. From baseline to post-treatment, following all CAP and CBT sessions, the patient showed a 98.5% reduction (RD) in pathological dissociation (*M*_pre_ = 19.7 vs. *M*_post_ = 0.3), no longer meeting criteria for D-PTSD. This was reflected by robust improvement in 11 clinically significant (cut-off score > 100) dissociative symptoms: derealization (*M*_pre_ = 27.5 vs. *M*_post_ = 0; RD = 100%), depersonalization (*M*_pre_ = 19.2 vs. *M*_post_ = 0; RD = 100%), flashbacks (*M*_pre_ = 24.2 vs. *M*_post_ = 0.8; RD = 96.7%), memory problems (*M*_pre_ = 52.5 vs. *M*_post_ = 1.7; RD = 96.8%), intrusive impulses (*M*_pre_ = 20.0 vs. *M*_post_ = 0; RD = 100%), trance (*M*_pre_ = 15.8 vs. *M*_post_ = 0; RD = 100%), time loss (*M*_pre_ = 45.0 vs. *M*_post_ = 0; RD = 100%), knowledge loss (*M*_pre_ = 26.0 vs. *M*_post_ = 0; RD = 100%), child voices (*M*_pre_ = 16.7 vs. *M*_post_ = 0; RD = 100%), internal voices (*M*_pre_ = 33.0 vs. *M*_post_ = 0; RD = 100%), and persecutory voices (*M*_pre_ = 30.0 vs. *M*_post_ = 0; RD = 100%). See Figure 3. She also exhibited marked decreases in cognitive distractibility (*M*_pre_ = 62.5 vs. *M*_post_ = 3.3; RD = 94.7%) and emotional suffering (*M*_pre_ = 47.5 vs. *M*_post_ = 2.5; RD = 94.7%), as measured by two response sets, the Cognitive Distraction Scale and Emotional Suffering Scale, both included in the MID.

Collectively, this led to anecdotal improvements in daily life activity, personal development, and overall wellbeing: “I've been able to do things I couldn't do before, like be home alone and with my daughters. I can do stuff around the house and be in public spaces. My fear and anxiety are gone. I don't feel like people are trying to hurt me. I feel more connected with myself and the world. I'm learning to accept my past trauma, without blame and judgment. This is something that changed my life.” She found interactional psychotherapy most beneficial in processing traumatic and relational memory: “I had all this gunk trapped inside that cannabis activated. Engaging with my therapist during sessions helped me resist, fully express, and clear that gunk – painful events I'd been carrying for a long time. I couldn't have done this alone.” The patient also described positive contributions from CBT: “It was extremely helpful to go back and explore my sessions. They were often challenging, but in a good way. Mentally understanding them gave me a lot of clarity and meaning.” Since treatment, the patient has engaged in traditional CBT, as needed, to address her complex trauma history. Remarkably, she has sustained improvements for over 2 years to date. This response was qualitatively described at 8-, 16-, and 24-months follow-up.

## Discussion

In this case of complex D-PTSD, CAP showed a striking and sustained reduction in pathological dissociation. This was accompanied by decreased cognitive distractibility and emotional suffering, as well as increased psychosocial functioning. Repeated sessions of CAP, paired with integrative CBT, likely account for the strength and durability of response. Moreover, with gains from CBT for PTSD lasting up to 12 months (36), ad hoc CBT may have prolonged the effects of therapy after treatment, with ongoing development of skills and knowledge overtime.

Notably, the patient described acute changes in oceanic boundlessness, ego-dissolution, and emotional breakthrough. These effects are surprising, given the low dose of cannabis used in each session. If cannabis and psychotherapy act synergistically, with therapy augmenting the response to cannabis, then its combined effect may explain the subjective changes. Irrespective, these facets of mysticism have been reported with high dose cannabis and overlap with those produced by classic psychedelics, including lysergic acid diethylamide (LSD), dimethyltryptamine (DMT), and psilocybin (37–41). They also reflect properties of ketamine, a dissociative agent, known to reduce self-referential awareness and induce feelings of unity, spirituality, and insight (42, 43). These parallels are intriguing, provided distinct mechanisms of action. Cannabis functions as a partial agonist at cannabinoid CB_1_ and CB_2_ receptors (20), whereas classic psychedelics generally activate serotonin (5–hydroxytryptamine, 5-HT) receptors, particularly 5-HT_2A_ (44, 45). Ketamine, on the other hand, is a non-competitive antagonist of N-methyl-D-aspartate (NMDA) glutamate receptors, with modulatory effects on neuroplasticity (46).

As psychedelic research on PTSD rapidly expands, the potential of cannabis as a novel pharmacotherapeutic is being questioned (47). This is evidenced by a growing body of literature, systematically reviewed in (27, 48–50). Nabilone, a synthetic cannabinoid that mimics the action of THC, is also being investigated for the condition. The drug shows promise for treating PTSD-related flashbacks and nightmares, with reported improvements in distressing dreams and sleep time (51–54). Notwithstanding, the literature on cannabis in PTSD is highly heterogeneous, stemming from observational and underpowered studies. Its application also remains controversial in humans, with a strong link between trauma and substance dependence (55). The use of adjunctive or combination psychotherapy has neither been explored; and data for D-PTSD is non-existent.

Future research on CAP stands to benefit from the psychedelic “highway,” as a feasible path toward clinical utility (56). This is evermore salient, given similarities between cannabis and psychedelics in public, commercial, and federal interests; the latter reflected by rising state and municipal legalization. Drawing parallels may additionally contribute to paradigm shifts in neuropsychiatry and drug development (57); specifically, looking beyond monoaminergic and glutamatergic systems to an endocannabinoid-based model of chronic stress pathology, aimed at neuromodulation. This may otherwise highlight a patient-specific model of trauma response and recovery.

Psychotherapy must also be considered. As a first-line treatment for PTSD, psychotherapy plays a key role in processing traumatic events, often through re-experiencing. It can also target more distressing ailments, such as guilt and shame, not readily addressed by normalizing neurochemical imbalances. In the present study, cannabis was paired with interactional psychotherapy, namely PSIP, to target complex relational trauma that manifested as fear, negative emotions, and detachment. It was additionally used to support inherent, self-correcting processes that arose during CAP. Learning how psychological interventions, like PSIP, maximize altered states of consciousness will be critical in characterizing mechanisms that lead to favorable outcomes. Hence, it is recommended that CAP be understood in the wider landscape of psychedelic-assisted therapy. For instance, understanding whether CAP is preferentially suited for treating D-PTSD, assuming that all assisted forms of psychedelics are available. A recent cross-sectional study, investigating expectations for CAP, showed comparable beliefs to psilocybin-assisted therapy among two samples of cannabis users (58). Participants believed that CAP, when administered at an ideal dose, could elicit mystical and emotional experiences, as well as alter dysfunctional attitudes. However, more data on CAP is patently needed to establish comparisons, specifically for D-PTSD. Finally, the role of “set and setting” should be examined. As with psychedelics (59), one's mindset and external environment, including the therapist, may interact to shape acute and long-term mental health outcomes.

## Limitations

This study has inherent limitations. It describes the history, symptoms, diagnosis, treatment, and follow-up of an individual patient, with no randomization, control, or blinding. Cannabis dosing was also subjectively variable, ranging from 6–10 mg per session. Moreover, it is unclear whether cannabis and psychotherapy were interdependent and necessary, the degree to which each produced clinical benefit, and how effective the treatment would have been with fewer or more sessions. Lastly, a directive, interactional approach to psychotherapy was employed, with an emphasis on autonomic and relational processing. This contrasts to other psychotherapeutic techniques, including cognitive, behavioral, and humanistic therapy. It also differs from gold-standard, evidence-based treatments for PTSD, namely CPT and PE, that are cognitive and exposure-based. Hence, it is premature to generalize the findings of this report. Nonetheless, this study represents the first data on CAP in D-PTSD, within a naturalistic, real-world setting. The results are more robust given the patient's clinical non-response to first- and second-line therapies, the complexity of this population, and its limited evidence base. Larger, well-controlled, and more diverse studies are required to explore potential underlying mechanisms, establish safety profiles and side effects, and assess therapeutic efficacy and effectiveness.

## Conclusions

This case highlights the potential of CAP in D-PTSD, with robust and sustained improvement in pathological dissociation. No adverse events were reported. Notably, subjective effects were comparable to those observed in psychedelic therapy, specifically oceanic boundlessness, ego dissolution, and emotional breakthrough. Further data is needed to explore, establish, and optimize CAP in D-PTSD, to determine the contexts and therapeutic frameworks it is best suited for, and to characterize its role in the current pharmacological toolbox.

## Data availability statement

The original contributions presented in the study are included in the article/supplementary material, further inquiries can be directed to the corresponding author.

## Ethics statement

Ethical review and approval was not required for the study on human participants in accordance with the local legislation and institutional requirements. The patients/participants provided their written informed consent to participate in this study. Written informed consent was obtained from the individual(s) for the publication of any potentially identifiable images or data included in this article.

## Author contributions

MK conceptualized the study and drafted the initial manuscript. AR supervised the work, created all figures, and drafted the final manuscript. MS contributed to the literature review and co-drafted the introduction. CM co-drafted the case presentation. AL co-drafted the results. LJ, AM, and RA contributed to the acquisition of data and critically revised the manuscript for intellectual content. PS, RR, and LA provided field expertise, interpretations of data, and substantial manuscript revisions. All authors have read and approved the final manuscript.
